# Prevalence and Characteristics of Itch and Pain in Patients Suffering from Chronic Hand Eczema

**DOI:** 10.3390/jcm12134198

**Published:** 2023-06-21

**Authors:** Adam Zalewski, Piotr K. Krajewski, Jacek C. Szepietowski

**Affiliations:** Department of Dermatology, Venereology and Allergology, Wroclaw Medical University, 50-368 Wroclaw, Poland

**Keywords:** hand eczema, itch, pain, chronic inflammation, inflammatory disease, quality of life, stigmatization

## Abstract

Background: Hand eczema (HE) is a frequent chronic inflammatory dermatosis. Itch and pain are considered two of the most common and burdensome symptoms of the disease. Yet, the data related to these symptoms are still limited. The aim of this study was to evaluate characteristics of itch and pain in adults suffering from HE. Methods: The study group comprised 100 adult HE patients. An original questionnaire designed by the authors was used to survey the patients. It included questions regarding demographic characteristics such as the duration of the disease, exacerbation count, past diagnostics and treatment, as well as atopic predispositions. Additionally, the itch and pain intensity (numerical rating scale—NRS) during ‘3 days prior to the study’ and the ‘entire disease’ period was implemented. The clinical assessment of the disease severity was performed according to two specific measurement instruments: Investigator Global Assessment for Chronic Hand Eczema (IGA-CHE) scale and Hand Eczema Severity Index (HECSI). To assess patient quality of life (QoL), the DLQI tool was used and to determine the level of stigmatization and for its impact on patients’ life the 6-Item Stigmatization Scale (6-ISS) was employed. Results: Within the period of 3 days prior to the examination, itch was reported by 81.0% of patients (*n* = 81), whereas 53.0% (*n* = 53) of them experienced pain. Both symptoms were reported more frequently in females (itch: *p* = 0.022; pain: *p* = 0.033). When sexes were compared, females reached higher scores in both IGA-CHE and HECSI. Itch and pain intensity correlated positively with disease severity. The intensity of itch and pain significantly influences HE patients’ QoL. A positive correlation between the 6-ISS score and the intensity of itch in the ‘last 3 days’ period was revealed (r = 0.221; *p* = 0.027). Conclusions: Itch and pain are common symptoms in HE patients, significantly contributing to the feeling of stigmatization. Providing characteristics of itch and pain may improve HE management. Symptom-decreasing treatment would definitely have a positive influence on patients’ well-being.

## 1. Introduction

Hand eczema (HE) is a recurrent inflammatory dermatosis with a high 1-year prevalence reaching 9.7% [1] and lifetime prevalence ranging from 11.3% to 20% [2,3,4,5]. The incidence rate of the disease is reported to be 5.5–8.8 per 1000 person years [1,4,6,7]. HE is also presented as a disease that affects many diverse aspects of patients’ life (i.e., physical, material, social and psychological) and impairs their quality of life (QoL) [3,8]. The term ’chronic’ is commonly used in relation to HE (chronic hand eczema; CHE) and it describes a condition which persists for at least 3 months or reoccurs for a minimum of two times in a 12-month period [4,9]. There is debate on the many factors that take part in CHE etiopathogenesis and the course of the disease. The etiology of HE is complex and depends on both endogenous (e.g., genetic) and exogenous (e.g., environmental or occupational) agents [8,10]. Endogenous variables are predominantly associated with the dysfunction of the skin barrier, which is characteristic of atopic dermatitis (AD) patients. An increased risk of developing a severe type of HE in this group of individuals is described, especially in coexistence with filaggrin mutation [11,12]. Exogenous factors contributing to HE development may be defined as forms of exposure leading to skin barrier deterioration. Among the most frequent, work in wet conditions (or water in general), irritants, toxic agents and mechanical irritation of the skin are suggested [7,13].

The most common primary lesions observed in HE patients are erythema, papules, oedema, vesicles or crusting. Additionally, signs of lichenification, areas of hyperkeratosis, scaling, fissures or erosions may appear. Manifestations of HE usually evolve in severity and clinical presentation over time. [8,14] Signs and complaints reported by patients predominantly include itching as well as pain, burning, stinging and mood and sleep disturbances. All of the above-mentioned complaints may lead to difficulties in everyday duties or the avoidance of social activities which involve hands [14,15,16]. Despite their high incidence, data related to itch and pain in HE are still lacking. A better overview and understanding of these symptoms may help physicians to improve diagnosis and management. It can also contribute to achieving better outcomes when it comes to increasing patients’ QoL.

Itch can be defined as an unpleasant sensation which leads to scratching. It can be classified as acute or chronic, depending on time criteria (less or more than 6 weeks, respectively) [17,18]. Four different types of itch can be distinguished: neuropathic (a result of central or peripheral nervous system damage), systemic (caused by systemic diseases such as infections or metabolic disorders), psychogenic (of psychiatric or psychosomatic origin) and dermatological (triggered by an invasion of exogenous substances causing an inflammatory response such as an insect bite, scabies infestation or sunburn, or occurring as a result of a various dermatoses, e.g., lichen planus). Additionally, a combination of these types is possible as well as the coexistence of more than one type in a single patient [19,20,21]. Itch occurs frequently not only in the course of HE, but also in other inflammatory dermatoses.

Pain itself may be classified in various ways; for example, acute and chronic. The latter is described to affect over 30% of people worldwide [22]. Another classification of pain distinguishes between nociceptive pain (a result of tissue injury or a stimuli that may potentially lead to tissue damage), neuropathic (caused by nerve injury or a disease affecting the nervous system, and may be commonly associated with numbness or allodynia) and nociplastic pain (triggered by a sensitized nervous system, without objective tissue or nerve damage) [22,23,24,25,26]. The mixed pain phenotype is being increasingly recognized by clinicians and researchers as a combination of the above-mentioned types [22]. In patients suffering from various dermatoses (such as HE, AD, psoriasis, hidradenitis suppurativa (HS)) pain is described as one of the most frequently occurring symptoms. It may be limited to skin lesions or generalized, often also manifesting as musculoskeletal pain [26]. 

Assessment of QoL and health-related QoL (HRQoL) has become one of the most important means used to evaluate the effects of interventions in various studies or the impact of the disease on patients’ well-being. The following types of HRQoL instruments can be distinguished: generic instruments—whose results may be compared across different disorders—such as EQ-5D, and disease-specific instruments—assessing the influence of a particular disease on QoL—namely the Quality of Life in Hand Eczema Questionnaire (QOLHEQ). Additionally, a skin-specific group is recognized, as represented by Dermatology Life Quality Index (DLQI) or Skindex-17 questionnaires [27]. Currently, there is no Polish version of QOLHEQ that is validated.

The aim of this study was to evaluate the prevalence and characteristics of itch and pain in a group of patients suffering from HE. 

## 2. Materials and Methods

### 2.1. Studied Group

A cross-sectional, prospective study was conducted on a group of 100 consecutive patients from an inpatient (hospital ward) and outpatient clinic at the Department of Dermatology, Venereology and Allergology in Wroclaw, Poland, between 1 February 2022 and 31 January 2023. All patients were recruited by the authors of this research. The whole studied population was diagnosed with chronic HE, based on the clinical picture and time criteria. Inclusion criteria comprised adult age (≥18 years old) and a course of disease persisting for over 3 months, enabling a chronic form of HE diagnosis. All patients with skin lesions that resembled CHE without a definite diagnosis or those waiting for the result of the biopsy were excluded from the study. An original survey, in the form of an investigation sheet, concerning group characteristics was prepared by the authors of the research prior to the study. The survey comprised questions regarding data related to the demographic data, the duration of the disease, exacerbation count, past diagnostics and treatment and atopic predispositions. Additionally, particular clinical features of the itch were investigated such as affected area (only within skin lesions vs. skin lesions and healthy skin). Patients were also asked to report where the skin lesions were located (only hands; hands and feet; hands, feet and other regions—disseminated lesions) and to declare whether they smoked tobacco or not (if the answer was positive, patients were asked to indicate the number of cigarettes smoked daily). Patients completed questionnaires concerning itch and pain assessment, as well as validated Polish versions of the instruments assessing quality of life (Dermatology Life Quality Index; DLQI) and stigmatization level (6-Item Stigmatization Scale; 6-ISS).

The study was approved by the local ethics committee (consent no. KB-234/2023) and written informed consent was obtained from all studied individuals. 

### 2.2. Disease Severity Assessment

The clinical assessment of the severity of the disease was performed according to English versions of two specific measurement instruments: Investigator Global Assessment for Chronic Hand Eczema (IGA-CHE) scale and Hand Eczema Severity Index (HECSI). All investigators were instructed and trained on their use prior to the study. 

IGA-CHE (physicians’ global assessment; PGA) distinguishes 5 categories of HE severity: ‘clear’, ‘almost clear’, ‘mild’, ‘moderate’ and ‘severe’ [28]. 

The HECSI is a tool employed to assess disease severity, combining the intensity, extent and clinical signs of HE. According to HECSI, both hands of the patient are divided into five areas: fingertips, fingers (except the fingertips), the palm of the hand, back of the hand and wrists. Within each area, the intensity of the following six clinical signs—erythema, induration/papulation vesicles, fissuring, scaling, and oedema—are graded on a scale as follows: 0 (no skin changes), 1 (mild disease), 2 (moderate disease), and 3 (severe disease). For each specific location, the total affected area (considering both hands) is given a score from 0 to 4 for the extent of clinical symptoms (0 = 0%, 1 = 1–25%, 2 = 26–50%, 3 = 51–75%, and 4 = 76–100%). Finally, the score given for the extent of clinical symptoms at each location is multiplied by the total sum of the intensity of each clinical feature and summated. The HECSI score ranges from 0 to 360 points (the maximum severity score) [29]. The following criteria for allocating patients into groups in relation to disease severity were used in our study: clear, 0; almost clear, 1–16; moderate, 17–37; severe, 38–116; very severe, ≥117 points [30]. 

### 2.3. Itch and Pain Assessment

Itch and pain intensity were evaluated using a numerical rating scale (NRS). All study participants were asked to assess the severity of the worst itch and pain during the last 3 days as well as the worst itch and pain during the entire period of the disease. The NRS is a unidimensional, brief symptom intensity measurement scale ranging from 0 (no itch/pain) to 10 (worst imaginable itch/pain). The scores of the itch NRS range from no (0 points), mild (1–3 points), moderate (4–6 points), severe (7–8 points), to very severe (≥9 points) [31]. In order to assess pain, the authors decided to use the following cut-off points for pain NRS, which were established at: ≤5—mild pain; >5–7—moderate pain; >7–10—severe pain, on the 0–10 rating scale [32]. 

### 2.4. QoL and Stigmatization Assessment

The validated Polish version of the DLQI questionnaire was implemented to assess issues related to quality of life [33]. DLQI is a dermatology-specific tool with a 1-week recall period, evaluating symptoms and feelings, daily activities, leisure, work and school aspects of life, relationships and side effects of treatment. It comprises 10 items, each scored from 0 to 3 points (0—‘not at all’; 1—‘a little’, 2—‘a lot’ 3—‘very much’). All individual scores are summed up and a total DLQI result is obtained, ranging 0–30 points. A score of 0–1 indicates no impact of the disease on QoL; a score of 2–5 points indicates a small impact; 6–10 points indicate a moderate impact; 11–20 points indicate a large impact; 21–30 points indicate an extremely large impact [33,34,35]. 

The 6-ISS (validated; in Polish) [36,37] was used to determine the level of stigmatization and its impact on patients’ life [38]. This instrument requires patients to answer 6 questions using one of four responses scored from 0 to 3 points (‘not at all’, ‘sometimes’, ‘very often’, and ‘always’). The higher the result, the greater the perception of stigmatization (ranging 0–18 points) [38]. 

### 2.5. Statistical Analysis

The statistical analysis of the acquired data was performed using IBM SPSS Statistics v. 26 (SPSS INC., Chicago, IL, USA) software. Firstly, all the data were assessed for parametric or non-parametric distribution using the Kolmogorov–Smirnov normality test. The minimum, maximum, mean, standard deviations and ranges were calculated. Based on the normality, analyzed quantitative variables were evaluated using the students *t* test or Mann–Whitney U test for parametric and non-parametric data, respectively. Depending on the normality, Spearman’s and Pearson’s correlations were used for the correlation assessments. For the qualitative data, the Chi^2^ test was used. Differences in analyzed data between more than two groups were evaluated, depending on normality, with ANOVA or the Kruskal–Wallis 1-way analysis of variance in ranks. A 2-sided *p* value of ≤ 0.05 was statistically significant.

## 3. Results

The study group consisted of 60 women (60.0%) and 40 men (40.0%), with an age range of 18–80 (mean 46.0 ± 17.23) years. The mean disease duration time was assessed at 42.5 months (SD = 60.84) and ranged from 3 to 396 months. The total exacerbation count at 12 months prior to the study reached 4.7 ± 3.6. Out of 100 participants, 71 (71.0%) were reported to have been treated because of HE in the past, with a predominance of females (*n* = 47; 78.3%) compared to males (*n* = 24; 60.0%). The difference was statistically significant (*p* = 0.048). Importantly, 28.0% of the studied group (*n* = 28) was treated systemically with glucocorticosteroids, methotrexate or alitretinoin. Atopic predispositions in the past (a history of asthma, allergic rhinitis, or atopic dermatitis) were indicated by 45 patients (45.0%). Moreover, 16 females (26.7%) and 12 (30.0%) males showed a correlation between occupation and disease occurrence. Only 27.0% (*n* = 27) of our patients were previously diagnosed with the use of patch tests (prior to the first visit at our department) and in 14.0% (*n* = 14) of all cases an allergic contact background was detected. Out of the whole population included in the research, 15.0% of the participants were active smokers. The difference between males and females was statistically significant (*p* = 0.022)—25.0% of males (*n* = 10), and 8.3% of females (*n* = 5) admitted to smoking. Additionally, the difference in the mean number of smoked cigarettes was statistically significant (1.98 ± 4.60 for males; 0.83 ± 2.94 for females; *p* = 0.029). All data concerning group characteristics are presented in Table 1. 

Considering the IGA-CHE, no patients were allocated in IGA-CHE group 0. Briefly, 15% of individuals (*n* = 15) belonged to IGA-CHE group 1, 25.0% (*n* = 25) belonged to IGA-CHE group 2, 37.0% (*n* = 37) belonged to IGA-CHE group 3, and 23.0% (*n* = 23) belonged to IGA-CHE group 4. Regarding the HECSI, the mean value was equal to 35.0 ± 27.8 points, with 29.3 ± 26.7 points for males and 38.8 ± 28.1 points for females. Table 2 presents the size of the particular HE severity groups, regarding the IGA-CHE, whereas Table 3 relates to the HECSI. When it comes to IGA-CHE scores for males and females, most of the men had scores falling within IGA groups 1 and 2 (*n* = 11; 27.5% in both groups), whereas women predominated in IGA group 3 (*n* = 28; 46.7%). No correlation between active smoking or the number of smoked cigarettes and disease severity was found.

### Characteristics of the Itch and Pain

Within 3 days prior to the examination, the majority, 81.0% (*n* = 81), of individuals reported itching, whereas 53.0% (*n* = 53) of patients experienced pain during the same period of time. During the ‘last 3 day’ period both symptoms were reported more frequently in females (pruritus: 53 females (88.3%) vs. 28 males (70%), *p* = 0.022; pain: 47 females (78.3%) vs. 16 males (40.0%), *p* = 0.033). During the whole disease period, 100% of the studied group reported itching, including both males and females. In the same period, pain was perceived by 53 females (88.3%) and 29 males (72.5%). For the whole studied population itch intensity was assessed to be 6.4 ± 2.7 points, for the entire disease period, which corresponds to ‘moderate’ itch, and for the ‘last 3 days’ period, it was assessed to be 3.9 ± 2.9 points, representing ‘mild’ itch (both measured with NRS). The pain severity for the entire disease and the ‘last 3 days’ period was rated as 4.6 ± 3.2 points and 2.6 ± 3.1 points (both corresponding to ‘mild’ pain), respectively. Evaluating outcomes with regard to the cut-off values, analyzing the entire disease period, the ‘very severe’ itch group was significantly more often represented by females in comparison to males (22 (36.7%) females vs. 5 (12.5%) males; *p* = 0.008), while in the ‘moderate’ itch group the predominance of males was observed (21 (52.5%) males vs. 19 (31.7%) females; *p* = 0.037). The results of the itch and pain assessment are included in Table 4 and Table 5, respectively. 

**Figure 1:**
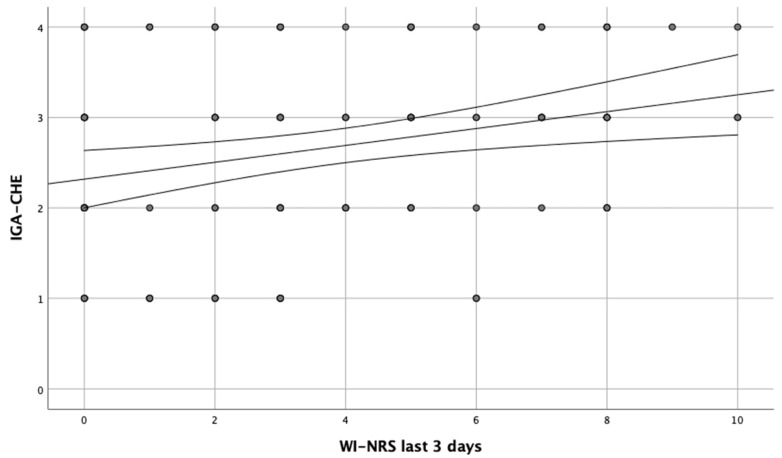
Correlation between worst itch (WI) in intensity of the last 3 days and IGA-CHE score.

**Figure 2:**
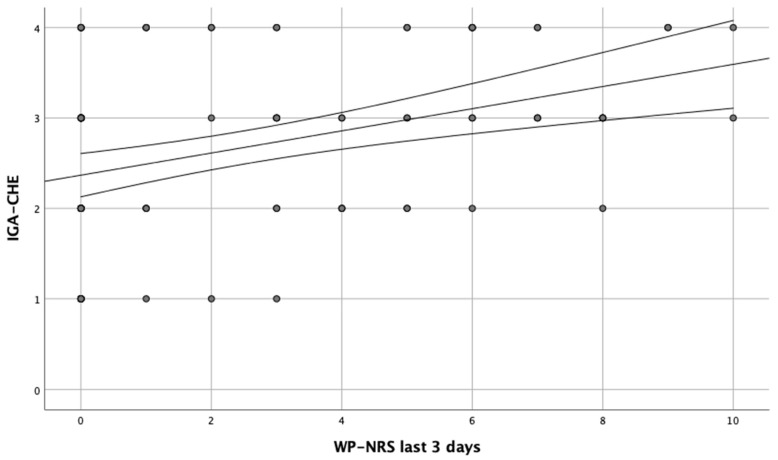
Correlation between worst pain (WP) in intensity of the last 3 days and IGA-CHE score.

A positive correlation between itch as well as pain intensity and IGA-CHE was observed, when these parameters 3 days prior to the examination period were taken into account (r = 0.261, *p* = 0.009; r = 0.377, *p* = 0.002, respectively) (Figure 1 and Figure 2). Additionally, similar correlations were found between itch and pain intensity during the entire disease period and IGA-CHE (r = 0.307, *p* = 0.002; r = 0.350, *p* < 0.001, respectively). Moreover, comparing the particular IGA-CHE severity groups, statistically significant differences in itch (*p* = 0.029) and pain (*p* = 0.002) intensity were found, only for the ‘last 3 days’ period. Results of the HESCI score also correlated with itch severity, in the entire disease period (r = 0.255, *p* = 0.01). No such correlation was found for the ‘last 3 days’ period. In the analysis of pain severity, a correlation with HECSI was found for both the ‘last 3 days’ and the entire disease period (r = 346, *p* < 0.001; r = 357, *p* < 0.001, respectively). No correlation between active smoking or the number of smoked cigarettes and itch or pain severity was found.

As for the localization of the lesions, in 65.0% (*n* = 65) of cases only hands were affected by eczematous lesions. Out of the whole population, 23.0% of patients reported foot skin involvement (*n* = 23). The remaining group (12.0%, *n* = 12) declared a disseminated form of the disease. Most of the investigated population (87.0%, *n* = 87) answered that itch was present only within skin lesions, whereas 13.0% of respondents experienced itching additionally in unaffected skin areas. No significant differences between sexes regarding the above-mentioned features were found. In all respondents reporting pain, it was localized only within skin lesions. 

Severity of both itch and pain correlated with reduced QoL measured with DLQI (Table 6).

Based on the level of stigmatization, a positive correlation between the 6-ISS score and the intensity of itching in the ‘last 3 days’ period was recognized (r = 0.221; *p* = 0.027). No correlation between the 6-ISS score and the intensity of itching in the ‘entire disease’ period, the intensity of pain in the ‘last 3 days’ period, or the intensity of pain in the ‘entire-disease’ period was found.

## 4. Discussion

According to the systematic review of Quaade et al. [39], one of the known risk factors of HE is being of a female sex which may be elucidated by various environmental factors. As a probable consequence, a higher incidence among women is reported. Additionally, a higher prevalence of atopic dermatitis in females is also observed [40] which corresponds with a higher rate of concomitant HE and atopic dermatitis among females. Within the group investigated in this research, the majority was constituted by females (60%), which may confirm the above-mentioned hypothesis. However, a difference regarding the prevalence of atopic predispositions between both sexes was not found. 

Sørensen et al. [41] indicated in their study that active tobacco smoking can increase the frequency of hand eczema, especially in high-risk occupations. This was explained by the delayed restoration of the broken skin barrier in smoking HE individuals [41]. Another meta-analysis, comprising 17 studies, revealed low-quality evidence that smoking is associated with a greater incidence of hand eczema (OR, 1.18; 95% CI, 1.09–1.26) [42]. Our study shows no correlation between active smoking or the number of smoked cigarettes and disease severity as well as symptom intensity.

Over one-third of HE cases in the general population are estimated to be moderate to severe [39]. In our study, the mean HESCI score for all participants allowed us to categorize the tested population as moderate HE patients (HECSI score = 35.0 ± 27.8 points). However, when the comparison between the sexes was taken into account, a difference in disease severity was visible: 29.3 ± 26.7 points for males, which corresponds to moderate HE, and 38.6 ± 28.1 points in females, representing severe HE. 

HE is described as one of the most frequently occurring diseases in patients suffering from itch [43]. Simultaneously, itch is also reported as one of the most common symptoms among HE patients, being reported by up to 78.1% of them [44,45,46]. In the study of Meding et al. [44], 50% of patients described the occurrence of itch as “frequent” and another one-third of the studied group reported it to be “occasional”. Yet, the data characterizing it are still insufficient. Itch is also defined as a burdensome symptom causing difficulties in daily functioning (touching and gripping objects), sleep disturbances, aesthetic issues, frustration and discomfort [16,45,47]. It is usually associated with disease exacerbation and the occurrence of other symptoms such as erythema, bleeding or scaling [47]. In 2014, Ruppert et al. [46] conducted a cross-sectional analysis evaluating factors associated with the presence and severity of itch among CHE patients. The results of their study demonstrated positive correlations with itch for specific age groups (17–25 and 26–45 years), which contrasted the outcomes of other reports, in which it was revealed thatthe older population was more prone to be affected by itch [48,49,50]. Furthermore, a correlation between sex and the presence of itch was not found, in contrast to most available data showing that females predominate among patients suffering from itch [51,52,53]. The results of our study also show the predominance of women in the itch-positive group (regarding itch at 3 days prior to the study and for the entire disease period; *p* = 0.002 and *p* = 0.004, respectively). When the entire disease period in the individuals studied in our research was considered, itch correlated positively with disease severity measured with the use of both of the following instruments: IGA-CHE and HESCI. These results are in line with those of Ruppert et al. [46] in which positive a correlation of itch with different CHE severity levels (moderate, severe, and very severe) was also observed [46]. 

Itch can be one of the various symptoms in patients with other dermatoses such as HS (acne inversa), psoriasis and atopic dermatitis [54,55,56]. Comparing itch-related data in various inflammatory dermatoses, the lack of research and reports concerning HE itch pathogenesis is visible. Considering the divisions of itch types, itching associated with HE could be classified into the group of dermatological itch (associated with chronic dermatoses). The hypothesis of pruritus of a mixed etiology, with a neurogenic component also seems plausible (taking into account the similarity to AD). The urgency for itch investigations to be conducted on HE patients is unquestionable. 

Another symptom of HE that is as yet not fully characterized is pain. In a study by Dibenedetti et al. [45], the prevalence of pain in patients with severe HE was assessed to be 36.0% ± 28.5%. Among the individuals in our group, 53.0% (*n* = 53) of patients reported pain within the last 3 days prior to the study (37 females, 61.7%; 16 males, 40.0%). In their research, Weigandt et al. [57] determined the mean value of pain severity (NRS) to be 1.94 ± 2.67 points, which corresponds with our results (Table 4). Data concerning etiology, pathogenesis and mechanisms connected with this topic are still unclear. Although pain at 3 days prior to the study period occurred statistically more frequent in women, our study showed no statistically significant difference in pain severity in a comparison between males and females, regarding that 3 days prior to the study and during the entire disease period. Pain intensity correlated positively with IGA-CHE and HESCI scores. In 2020, Passlov et al. [58] conducted a study assessing the influence of HE on hand strength and dexterity. One of the measured parameters was pain level. Researchers observed a strong correlation between perceived disability to perform activities of daily living and pain. Their results indicated that pain experienced by HE patients may be a major limiting factor, regarding patients’ daily life [58]. Depending on typical features of HE such as redness, scaling, edema, vesicles, hyperkeratosis, fissures and erosions, a hypothesis of the nociceptive origin of HE-related pain may be proposed [15]. Nevertheless, the neuropathic pain pathway should also be considered and more research on this topic is urgently required. Pain is a symptom of special significance not only in HE individuals. Patients suffering from HS, psoriasis, and AD report pain as a common symptom of their diseases [59,60,61].

Quality of life and its improvement are some of the most frequently discussed issues in modern medicine and patient-oriented management. Alongside AD, moderate-to-severe HE is proven to have a stronger impact on QoL compared to most other chronic conditions [62]. A significant impairment in quality of life was observed even in patients with low HECSI scores (low severity of HE) [63]. Our study additionally indicates that the severity of the itch and pain also influences significantly HE patients’ QoL. What can positively influence the QoL of HE patients is proper education concerning the course of the disease. In the study of Rönsch et al. [64] special attention was focused on the need to receive appropriate and complete information from a physician. Patients also reported the high value of caregivers who listen to them [64]. 

Our study has several limitations that should be considered. The extent of the study involved only one center from Wroclaw, Lower Silesia, Poland, which reduces the variability of the population. The small sample size is also worth mentioning. In the future, a multicenter study involving patients from different geographical regions would be of special importance. So far, our group focuses neither on the pathogenesis of itch and pain, nor the pathways responsible for transmitting them, which would be crucial for subsequent investigations. Nevertheless, it should be emphasized that our work extends the current knowledge on subjective symptoms in HE patients.

## 5. Conclusions

Because of the wide range of etiological factors and clinical pictures, HE remains a disease entity which should constantly be investigated. Being one of the most frequently reported HE complaints, itch influences all aspects of patients’ life. Concerning the data suggesting that up to 81.0% of individuals suffer form HE, itch correlates with disease severity and is often associated with HE exacerbations. Our study shows that itch is reported more frequently by females (88.3% vs. 70.0%), concerning that at the ‘3-days-prior to the study’ period. Its prevalence was higher than the incidence of pain within a comparable period (81.0% vs. 53.0%). Pain in HE is a symptom which requires more attention and is often underestimated. Pain, similarly to itch, dominates among females. Our study demonstrates also that pain intensity correlates positively with the severity of the disease. Both itch and pain are major symptoms associated with HE, impairing patients’ well-being. Developing data characterizing them may help both physicians in improving their management and scientists in creating new therapeutic options. Symptom-decreasing or even liberating treatment would definitely have a positive influence on patients’ QoL and stigmatization level.

## Figures and Tables

**Table 1:** Demographic characteristics, risk factors, previous treatment and lesion location. Gender division has been applied, with the indication of the statistical significance.

Characteristics	Whole Population (*n* = 100)	Females (*n* = 60)	Males (*n* = 40)	*p*
Age, years (mean ± SD)	46.0 ± 17.23	46.6 ± 18.27	36.9 ± 13.2	NS
Disease duration, months (mean ± SD)	42.5 ± 60.84	30.85 ± 40.34	27.7 ± 7.1	NS
Exacerbation count in last 12 months (mean ± SD)	4.67 ± 3.55	4.72 ± 2.98	4.60 ± 4.31	NS
Previous treatment	71 (71.0%)	47 (78.3%)	24 (60.0%)	0.048
Systemic treatment	28 (28.0%)	17 (28.3%)	11 (27.5%)	NS
History of atopy/allergy	45 (45.0%)	26 (43.3%)	19 (47.5%)	NS
Observed correlation between disease and occupation	28 (28.0%)	16 (26.7%)	12 (30.0%)	NS
Diagnosed allergic contact background	14 (14.0%)	8 (13.3%)	6 (15.0%)	NS
Previous patch testing	27 (27.0%)	13 (21.7%)	14 (35.0%)	NS
Smokers	15 (15.0%)	5 (8.3%)	10 (25.0%)	0.022
Cigarettes count, number (mean ± SD)	1.29 ± 3.71	0.83 ± 2.94	1.98 ± 4.60	0.029
Lesion location				
Only hands	65 (65.0%)	38 (63.3%)	27 (67.5%)	NS
Hands and feet	23 (23.0%)	15 (25.0%)	8 (20.0%)	NS
Disseminated lesions	12 (12.0%)	7 (11.7%)	(12.5%)	NS

NS—not significant.

**Table 2:** Prevalence of individual IGA-CHE severity groups among HE patients, considering gender division.

IGA-CHE Severity Group	Total Group Size/ Frequency (*n* = 100)	Females (*n* = 60)	Males (*n* = 40)	*p*
0	0 (0.0%)	0 (0.0%)	0 (0.0%)	NS
1	15 (15.0%)	4 (6.7%)	11 (27.5%)	0.004
2	25 (25.0%)	14 (23.3%)	11 (27.5%)	NS
3	37 (37.0%)	28 (46.7%)	9 (22.5%)	0.014
4	23 (23.0%)	14 (23.3%)	9 (22.5%)	NS

NS—not significant.

**Table 3:** Prevalence of individual HECSI severity groups among HE patients, considering gender division.

HECSI Severity Group	Total Group Size/ Frequency (*n* = 100)	Females (*n* = 60)	Males (*n* = 40)	*p*
clear	0 (0.0%)	0 (0.0%)	0 (0.0%)	NS
almost clear	34 (34.0%)	15 (25.0%)	19 (47.5%)	NS
moderate	27 (27.0%)	20 (33.3%)	7 (17.5%)	NS
severe	37 (37.0%)	23 (38.3%)	14 (35.0%)	NS
very severe	2 (2.0%)	2 (3.3%)	0 (0.0%)	NS
mean HECSI value	35.0 ± 27.8	38.8 ± 28.1	29.3 ± 26.7	0.037

NS—not significant.

**Table 4:** Results of itch assessment, considering ‘last 3 days’ period and entire disease period with gender division.

	Whole Population (*n* = 100)	Females (*n* = 60)	Males (*n* = 40)	*p*
Itch in last 3 days				
mean maximum itch severity, points (mean ± SD)	3.88 ± 2.93	4.77 ± 2.90	2.55 ± 2.45	0.002
no (0 points)	19 (19.0%)	7 (11.7%)	12 (30.0%)	NS
mild (1–3 points)	17 (17.0%)	6 (10.0%)	11 (27.5%)	0.003
moderate (4–6 points)	40 (40.0%)	27 (45.0%)	13 (32.5%)	NS
severe (7–8 points)	20 (20.0%)	16 (26.7%)	4 (10.0%)	NS
very severe (≥9 points)	4 (4.0%)	4 (6.7%)	0 (0.0%)	NS
Itch during the whole disease period				
maximum itch severity, points (mean ± SD)	6.35 ± 2.65	6.97 ± 2.65	5.43 ± 2.41	0.004
no (0 points)	0 (0.0%)	0 (0.0%)	0 (0.0%)	NS
mild (1–3 points)	7 (7.0%)	3 (5.0%)	4 (10.0%)	NS
moderate (4–6 points)	40 (40.0%)	19 (31.7%)	21 (52.5%)	0.037
severe (7–8 points)	26 (26.0%)	16 (26.7%)	10 (25.0%)	NS
very severe (≥9 points)	27 (27.0%)	22 (36.7%)	5 (12.5%)	0.008

NS—not significant.

**Table 5:** Results of pain assessment, considering ‘last 3 days’ period and entire disease period with gender division.

	Whole Population (*n* = 100)	Females (*n* = 60)	Males (*n* = 40)	*p*
Pain in last 3 days				
Maximum pain severity, points (mean ± SD)	2.55 ± 3.06	3.40 ± 3.35	1.27 ± 2.01	0.005
no pain	47 (47.0%)	23 (38.3%)	24 (60.0%)	NS
mild (1–5 points)	38 (38.0%)	23 (38.3%)	15 (37.5)	NS
moderate (>5–7 points)	11 (11.0%)	10 (16.7%)	1 (2.5%)	NS
severe (>7–10 points)	4 (4.0%)	4 (6.7%)	0 (0.0%)	NS
Pain during the whole disease				
Maximum pain severity, points (mean ± SD)	4.59 ± 3.21	5.33 ± 3.27	3.48 ± 2.81	0.005
no pain	18 (18.0%)	7 (11.7%)	11 (27.5%)	NS
mild (1–5 points)	51 (51.0%)	29 (48.3%)	22 (55.0%)	NS
moderate (>5–7 points)	18 (18.0%)	12 (20.0%)	6 (15.0%)	NS
severe (>7–10 points)	13 (13.0%)	12 (20.0%)	1 (2.5%)	NS

NS—not significant.

**Table 6:** Correlations between itch/pain severity and DLQI.

	Correlation with DLQI (r)	*p*
Itch within last 3 days	0.436	<0.001
Pain within last 3 days	0.305	0.002
Itch during the whole disease	0.255	0.01
Pain during the whole disease	0.347	<0.001

## Data Availability

The datasets generated and analyzed in the current study are available from the corresponding author upon reasonable request.

## References

[1] Meding B., Järvholm B. (2002). Hand Eczema in Swedish Adults—Changes in Prevalence between 1983 and 1996. J. Investig. Dermatol..

[2] Vindenes H.K., Svanes C., Lygre S.H.L., Hollund B.-E., Langhammer A., Bertelsen R.J. (2017). Prevalence of, and Work-Related Risk Factors for, Hand Eczema in a Norwegian General Population (The HUNT Study). Contact Dermat..

[3] Maden S., Ozbagcivan O., Onur Aysevener B.E., Aktan S. (2021). Quality of Life, Anxiety, Depression, Social Anxiety and Avoidance in Patients with Chronic Hand Eczema. Ital. J. Dermatol. Venereol..

[4] Thyssen J.P., Johansen J.D., Linneberg A., Menné T. (2010). The Epidemiology of Hand Eczema in the General Population—Prevalence and Main Findings. Contact Dermat..

[5] Worm M., Thyssen J.P., Schliemann S., Bauer A., Shi V.Y., Ehst B., Tillmann S., Korn S., Resen K., Agner T. (2022). The Pan-JAK Inhibitor Delgocitinib in a Cream Formulation Demonstrates Dose Response in Chronic Hand Eczema in a 16-Week Randomized Phase IIb Trial. Br. J. Dermatol..

[6] Diepgen T.L., Andersen K.E., Brandao F.M., Bruze M., Bruynzeel D.P., Frosch P., Gonçalo M., Goossens A., Le Coz C.J., Rustemeyer T. (2009). Hand Eczema Classification: A Cross-Sectional, Multicentre Study of the Aetiology and Morphology of Hand Eczema. Br. J. Dermatol..

[7] Lerbaek A., Kyvik K.O., Ravn H., Menné T., Agner T. (2007). Incidence of Hand Eczema in a Population-Based Twin Cohort: Genetic and Environmental Risk Factors. Br. J. Dermatol..

[8] Coenraads P.-J. (2012). Hand Eczema. N. Engl. J. Med..

[9] Mowad C.M., Anderson B., Scheinman P., Pootongkam S., Nedorost S., Brod B. (2016). Allergic Contact Dermatitis: Patient Diagnosis and Evaluation. J. Am. Acad. Dermatol..

[10] Coenraads P.-J. (2007). Hand Eczema Is Common and Multifactorial. J. Investig. Dermatol..

[11] Heede N.G., Thuesen B.H., Thyssen J.P., Linneberg A., Szecsi P.B., Stender S., Menné T., Johansen J.D. (2017). Hand Eczema, Atopic Dermatitis and Filaggrin Mutations in Adult Danes: A Registry-Based Study Assessing Risk of Disability Pension. Contact Dermat..

[12] Visser M.J., Landeck L., Campbell L.E., McLean W.H.I., Weidinger S., Calkoen F., John S.M., Kezic S. (2013). Impact of Atopic Dermatitis and Loss-of-Function Mutations in the Filaggrin Gene on the Development of Occupational Irritant Contact Dermatitis. Br. J. Dermatol..

[13] Bryld L.E., Hindsberger C., Kyvik K.O., Agner T., Menné T. (2003). Risk Factors Influencing the Development of Hand Eczema in a Population-Based Twin Sample. Br. J. Dermatol..

[14] Thyssen J.P., Schuttelaar M.L.A., Alfonso J.H., Andersen K.E., Angelova-Fischer I., Arents B.W.M., Bauer A., Brans R., Cannavo A., Christoffers W.A. (2022). Guidelines for Diagnosis, Prevention, and Treatment of Hand Eczema. Contact Dermat..

[15] Agner T., Elsner P. (2020). Hand Eczema: Epidemiology, Prognosis and Prevention. J. Eur. Acad. Dermatol. Venereol..

[16] Moberg C., Alderling M., Meding B. (2009). Hand Eczema and Quality of Life: A Population-Based Study. Br. J. Dermatol..

[17] Ständer S., Weisshaar E., Mettang T., Szepietowski J.C., Carstens E., Ikoma A., Bergasa N.V., Gieler U., Misery L., Wallengren J. (2007). Clinical Classification of Itch: A Position Paper of the International Forum for the Study of Itch. Acta Derm. Venereol..

[18] Reszke R., Szepietowski J.C. (2020). Itch and Psyche: Bilateral Associations. Acta Derm. Venereol..

[19] Kahremany S., Hofmann L., Harari M., Gruzman A., Cohen G. (2021). Pruritus in Psoriasis and Atopic Dermatitis: Current Treatments and New Perspectives. Pharmacol. Rep..

[20] Tivoli Y.A., Rubenstein R.M. (2009). Pruritus. J. Clin. Aesthet. Dermatol..

[21] Ständer S. (2016). Classification of itch. Itch-Management in Clinical Practice.

[22] Cohen S.P., Vase L., Hooten W.M. (2021). Chronic Pain: An Update on Burden, Best Practices, and New Advances. Lancet.

[23] Nalamachu S. (2013). An Overview of Pain Management: The Clinical Efficacy and Value of Treatment. Am. J. Manag. Care.

[24] Finnerup N.B., Haroutounian S., Kamerman P., Baron R., Bennett D.L.H., Bouhassira D., Cruccu G., Freeman R., Hansson P., Nurmikko T. (2016). Neuropathic Pain: An Updated Grading System for Research and Clinical Practice. Pain.

[25] Saavedra-Hernández M., Castro-Sánchez A.M., Cuesta-Vargas A.I., Cleland J.A., Fernández-de-las-Peñas C., Arroyo-Morales M. (2012). The Contribution of Previous Episodes of Pain, Pain Intensity, Physical Impairment, and Pain-Related Fear to Disability in Patients with Chronic Mechanical Neck Pain. Am. J. Phys. Med. Rehabil..

[26] Pithadia D.J., Reynolds K.A., Lee E.B., Wu J.J. (2019). Psoriasis-Associated Cutaneous Pain: Etiology, Assessment, Impact, and Management. J. Dermatol. Treat..

[27] Ofenloch R.F., Weisshaar E., Dumke A.-K., Molin S., Diepgen T.L., Apfelbacher C. (2014). The Quality of Life in Hand Eczema Questionnaire (QOLHEQ): Validation of the German Version of a New Disease-Specific Measure of Quality of Life for Patients with Hand Eczema. Br. J. Dermatol..

[28] Ruzicka T., Lynde C.W., Jemec G.B.E., Diepgen T., Berth-Jones J., Coenraads P.J., Kaszuba A., Bissonnette R., Varjonen E., Holló P. (2008). Efficacy and Safety of Oral Alitretinoin (9-Cis Retinoic Acid) in Patients with Severe Chronic Hand Eczema Refractory to Topical Corticosteroids: Results of a Randomized, Double-Blind, Placebo-Controlled, Multicentre Trial. Br. J. Dermatol..

[29] Held E., Skoet R., Johansen J.D., Agner T. (2005). The Hand Eczema Severity Index (HECSI): A Scoring System for Clinical Assessment of Hand Eczema. A Study of Inter- and Intraobserver Reliability. Br. J. Dermatol..

[30] Oosterhaven J.A.F., Schuttelaar M.L.A. (2020). Responsiveness and Interpretability of the Hand Eczema Severity Index. Br. J. Dermatol..

[31] Cheung H.N., Chan Y.S., Hsiung N.H. (2021). Validation of the 5-D Itch Scale in Three Ethnic Groups and Exploring Optimal Cutoff Values Using the Itch Numerical Rating Scale. Biomed. Res. Int..

[32] Chien C.-W., Bagraith K.S., Khan A., Deen M., Syu J.-J., Strong J. (2017). Establishment of Cutpoints to Categorize the Severity of Chronic Pain Using Composite Ratings with Rasch Analysis. Eur. J. Pain.

[33] Szepietowski J.C., Salomon J., Finlay A.Y. Dermatology Life Quality Index (DLQI): Polish Version. Proceedings of the 11th International Congress European Society for Dermatology and Psychiatry.

[34] Finlay A.Y., Khan G.K. (1994). Dermatology Life Quality Index (DLQI)—A Simple Practical Measure for Routine Clinical Use. Clin. Exp. Dermatol..

[35] Barrett A., Hahn-Pedersen J., Kragh N., Evans E., Gnanasakthy A. (2019). Patient-Reported Outcome Measures in Atopic Dermatitis and Chronic Hand Eczema in Adults. Patient.

[36] Masnari O., Landolt M.A., Roessler J., Weingaertner S.K., Neuhaus K., Meuli M., Schiestl C. (2012). Self- and Parent-Perceived Stigmatisation in Children and Adolescents with Congenital or Acquired Facial Differences. J. Plast. Reconstr. Aesthetic Surg..

[37] Hrehorów E., Szepietowski J., Reich A., Evers A.W.M., Ginsburg I.H. (2006). Instruments for Stigmatization Evaluation in Patients Suffering from Psoriasis: Polish Language Versions. Dermatol. Klin..

[38] Dimitrov D., Szepietowski J.C. (2017). Instruments to Assess Stigmatization in Dermatology. Postępy Hig. Med. Doświadczalnej.

[39] Quaade A.S., Simonsen A.B., Halling A.-S., Thyssen J.P., Johansen J.D. (2021). Prevalence, Incidence, and Severity of Hand Eczema in the General Population—A Systematic Review and Meta-Analysis. Contact Dermat..

[40] Schultz Larsen F., Diepgen T., Svensson A. (1996). The Occurrence of Atopic Dermatitis in North Europe: An International Questionnaire Study. J. Am. Acad. Dermatol..

[41] Sørensen J.A., Clemmensen K.K., Nixon R.L., Diepgen T.L., Agner T. (2015). Tobacco Smoking and Hand Eczema—Is There an Association?. Contact Dermat..

[42] Loman L., Brands M.J., Massella Patsea A.A.L., Politiek K., Arents B.W.M., Schuttelaar M.L.A. (2022). Lifestyle Factors and Hand Eczema: A Systematic Review and Meta-Analysis of Observational Studies. Contact Dermat..

[43] Park S.-M., Kim J.-M., Kim G.-W., Kim W.-J., Kim H.-S., Ko H.-C., Kim M.-B., Kim B.-S. (2017). Assessment of Itch and Sensory Characteristics in Patients with Hand Eczema. Eur. J. Dermatol..

[44] Meding B., Swanbeck G. (1990). Consequences of Having Hand Eczema. Contact Dermat..

[45] Dibenedetti D., Baranowski E., Zelt S., Reynolds M., Sherrill B. (2015). Assessing United States Patient and Dermatologist Experiences with Severe Chronic Hand Eczema. J. Clin. Aesthet. Dermatol..

[46] Ruppert L., Apfelbacher C., Molin S., Bauer A., Mahler V., Schmitt J., Elsner P., Diepgen T.L., Weisshaar E. (2014). Itching in Patients with Chronic Hand Eczema: Data from the CARPE Registry. Dermatology.

[47] Grant L., Seiding Larsen L., Burrows K., Belsito D.V., Weisshaar E., Diepgen T., Hahn-Pedersen J., Sørensen O.E., Arbuckle R. (2020). Development of a Conceptual Model of Chronic Hand Eczema (CHE) Based on Qualitative Interviews with Patients and Expert Dermatologists. Adv. Ther..

[48] Sommer F., Hensen P., Böckenholt B., Metze D., Luger T.A., Ständer S. (2007). Underlying Diseases and Co-Factors in Patients with Severe Chronic Pruritus: A 3-Year Retrospective Study. Acta Derm. Venereol..

[49] Weisshaar E., Apfelbacher C., Jäger G., Zimmermann E., Bruckner T., Diepgen T.L., Gollnick H. (2006). Pruritus as a Leading Symptom: Clinical Characteristics and Quality of Life in German and Ugandan Patients. Br. J. Dermatol..

[50] Matterne U., Apfelbacher C.J., Loerbroks A., Schwarzer T., Büttner M., Ofenloch R., Diepgen T.L., Weisshaar E. (2011). Prevalence, Correlates and Characteristics of Chronic Pruritus: A Population-Based Cross-Sectional Study. Acta Derm. Venereol..

[51] Ständer S., Stumpf A., Osada N., Wilp S., Chatzigeorgakidis E., Pfleiderer B. (2013). Gender Differences in Chronic Pruritus: Women Present Different Morbidity, More Scratch Lesions and Higher Burden. Br. J. Dermatol..

[52] Weisshaar E., Dalgard F. (2009). Epidemiology of Itch: Adding to the Burden of Skin Morbidity. Acta Derm. Venereol..

[53] Dalgard F., Svensson A., Holm J.Ø., Sundby J. (2004). Self-Reported Skin Morbidity in Oslo. Associations with Sociodemographic Factors among Adults in a Cross-Sectional Study. Br. J. Dermatol..

[54] Agarwal P., Lunge S.B., Shetty N.S., Karagaiah P., Daveluy S., Ortega-Loayza A.G., Tzellos T., Szepietowski J.C., Zouboulis C.C., Grabbe S. (2022). Itch in Hidradenitis Suppurativa/Acne Inversa: A Systematic Review. J. Clin. Med..

[55] Szepietowski J.C., Reich A. (2016). Pruritus in Psoriasis: An Update. Eur. J. Pain.

[56] Mollanazar N.K., Smith P.K., Yosipovitch G. (2016). Mediators of Chronic Pruritus in Atopic Dermatitis: Getting the Itch Out?. Clin. Rev. Allergy Immunol..

[57] Weigandt W.A., Schardt Y., Bruch A., Herr R., Goebeler M., Benecke J., Schmieder A. (2023). Impact of an EHealth Smartphone App on Quality of Life and Clinical Outcome of Patients with Hand and Foot Eczema: Prospective Randomized Controlled Intervention Study. JMIR mHealth uHealth.

[58] Passlov H.M., Pontén A., Björk J., Rosén B., Bruze M., Svedman C., Isaksson M. (2020). Hand Strength and Dexterity in Individuals with Hand Eczema. J. Eur. Acad. Dermatol. Venereol..

[59] Matusiak Ł., Szczęch J., Kaaz K., Lelonek E., Szepietowski J.C. (2018). Clinical Characteristics of Pruritus and Pain in Patients with Hidradenitis Suppurativa. Acta Derm. Venereol..

[60] Ljosaa T.M., Rustoen T., Mörk C., Stubhaug A., Miaskowski C., Paul S.M., Wahl A.K. (2010). Skin Pain and Discomfort in Psoriasis: An Exploratory Study of Symptom Prevalence and Characteristics. Acta Derm. Venereol..

[61] Li J.-X., Dong R.-J., Zeng Y.-P. (2021). Characteristics, Mechanism, and Management of Pain in Atopic Dermatitis: A Literature Review. Clin. Transl. Allergy.

[62] Capucci S., Hahn-Pedersen J., Vilsbøll A., Kragh N. (2020). Impact of Atopic Dermatitis and Chronic Hand Eczema on Quality of Life Compared with Other Chronic Diseases. Dermatitis.

[63] Charan U.P., Peter C.V.D., Pulimood S.A. (2013). Impact of Hand Eczema Severity on Quality of Life. Indian Dermatol. Online J..

[64] Rönsch H., Schiffers F., Ofenloch R., Weisshaar E., Buse A.S., Hansen A., John S.M., Giménez Arnau A.M., Pesqué D., Agner T. (2023). Chronic Hand Eczema in Europe: Patient Experiences and Perspectives (CHEPEP) in Qualitative Interviews. J. Eur. Acad. Dermatol. Venereol..

